# Cigarette smokers’ views on their habit and the causes of their illness following lung cancer diagnosis: a clinical-qualitative study

**DOI:** 10.1590/S1516-31802006000300003

**Published:** 2006-05-04

**Authors:** Olívia Meira Dias, Egberto Ribeiro Turato

**Keywords:** Lung neoplasms, Psychological adaptation, Qualitative research, Smoking, Tobacco, Neoplasias pulmonares, Adaptação psicológica, Pesquisa qualitativa, Tabagismo, Tabaco

## Abstract

**CONTEXT AND OBJECTIVE:**

Lung cancer is the commonest malignant tumor and is increasing in incidence by 2% a year. In 90% of diagnosed cases, it is associated with tobacco product consumption. It is the greatest cause of mortality among cancer types in Brazil. Knowledge of patients’ psychological representations is needed for evaluating treatments and educating patients. The aim here was to interpret how smokers with lung cancer interpret the possible causes of their illness and to understand their perceptions regarding cigarette use.

**DESIGN AND SETTING:**

Clinical-qualitative study (exploratory, non-experimental) at the Pulmonary Disease Service, General Hospital, Universidade Estadual de Campinas.

**METHODS:**

An intentional small sample of cancer inpatients was recruited. The group was closed with 11 subjects, following attainment of data saturation from interviews. These interviews were semi-directed, with in-depth open-ended questions on interviewees’ observations, applied in a confidential setting using a tape recorder. Interviewees’ responses were categorized using qualitative content analysis and the results were assessed using interdisciplinary theoretical concepts, particularly from medical psychology.

**RESULTS:**

Six males and five females aged between 46 and 68 years who presented diverse clinical conditions were interviewed.

**CONCLUSIONS:**

A broader approach towards the psychological comprehension of such patients is needed, considering that cigarette consumption involves conscious and unconscious motivations, sociocultural and educational factors, the glamour of tobacco advertising, and problems with psychophysical dependence. Such an approach would avoid the perception among patients that the healthcare team are “inquisitors”. This would lead to better adherence to treatment and better quality of life.

## RESUMO

**CONTEXTO E OBJETIVO:**

O câncer de pulmão é o mais comum dos tumores malignos, apresentando aumento de 2% ao ano em sua incidência mundial. Em 90% dos casos diagnosticados, está associado ao consumo de derivados de tabaco. No Brasil, é o câncer que mais faz vítimas fatais. É crucial aos médicos conhecer as representações psicológicas desses pacientes para preconizar tratamentos e educá-los. O objetivo deste trabalho é interpretar significados que fumantes com câncer de pulmão atribuem a possíveis causas da doença, bem como compreender percepções relacionadas ao uso do cigarro.

**TIPO DE ESTUDO E LOCAL:**

Desenho clínico-qualitativo (exploratório, não-experimental) realizado no Serviço de Pneumologia do Hospital das Clínicas da Universidade Estadual de Campinas, Campinas, São Paulo, Brasil.

**MÉTODOS:**

Amostra propositalmente pequena de pacientes internados com câncer; grupo fechado com 11 sujeitos quando ocorrida a saturação de informações das entrevistas. Entrevista semidirigida de questões abertas (entrevista em profundidade na observação acurada dos entrevistados) contendo poucos tópicos foi aplicada em ambiente confidencial, usando gravador de fita cassete. Após categorização do conjunto das falas, usando análise qualitativa de conteúdo, a discussão dos resultados empregou conceitos teóricos interdisciplinares, especialmente da psicologia médica.

**RESULTADOS:**

Entrevistamos seis homens e cinco mulheres, com idades variando entre 46 e 68 anos; sujeitos apresentando condições clínicas diversas.

**CONCLUSÃO:**

A compreensão psicológica desses pacientes exige uma abordagem mais ampla, considerando que o consumo de cigarros envolve motivações conscientes e inconscientes, fatores socioculturais e educacionais, glamourosa propaganda do tabaco e problemas de dependência físico-psíquica. Também evitaria o surgimento de figuras percebidas pelos pacientes como “inquisidores” dentre a equipe de saúde. Estas novas condições levariam à maior adesão ao tratamento e melhor qualidade de vida.

## INTRODUCTION

According to the Brazilian National Cancer Institute, lung cancer is the greatest cause of mortality among cancer types in Brazil. It is also the most common malignant tumor, and its worldwide incidence is increasing at a rate of 2% a year. In 90% of the cases diagnosed, it is associated with the consumption of tobacco products.^1^ According to the American Cancer Society, there will be more than 172,000 new cases of lung cancer during 2005, of which about 54% will be among males and 46% among females. Lung cancer will account for about 13% of all new cancer cases and it will mainly occur among people diagnosed at the age of 70 years or over.^2^

The chance for a man to develop lung cancer is 1 in 13 and for a woman, 1 in 18, among the whole population, whether they are smokers or not. Lung cancer is the leading cause of deaths due to cancer among both men and women. It has been estimated that there will be more than 163,000 deaths from lung cancer in 2005, accounting for around 28% of all deaths due to cancer. Out of the large number of people diagnosed with this type of cancer, there are only about 330,000 long-term survivors. Nearly 60% of the individuals with lung cancer die within 1 year following its diagnosis, and nearly 75% die within 2 years. This situation has not improved over the last 10 years.^2^

Despite the improvements that have been made in medicine, attempts at finding curative treatments have met little success. Stopping smoking and efforts towards controlling tobacco use remain the most important factors in attempts to reduce the incidence of this disease.^3^

On the other hand, failure to detect and treat the distress puts at risk the outcomes from cancer therapy, thereby decreasing patients’ quality of life and increasing healthcare costs. Zabora et al.^4^ investigated the prevalence of psychological distress among a large sample of cancer patients, and determined the variations in distress among 14 cancer diagnoses. They found that the overall prevalence of distress was 35.1%, while the figure for lung cancer cases was 43.4%. Sarna et al.^5^ found serious disruptions in the psychosocial aspects of quality of life among women with lung cancer, particularly depressed moods and negative attitudes towards the illness among the younger patients.

According to Kahana,^6^ a physician who had lung cancer, living with an incurable disease like this meant having to face the idea of dying. This author believed that, for religious people, the concept of an afterlife might or might not provide comfort, but for an atheist like himself, there was the blackness of the void. If in childhood there was the belief that one would live forever, he now found himself becoming a trusting child again, at least transiently.

To explore whether patients with life-threatening illnesses considered that their experiences signified a spiritual need, Murray et al.^7^ conducted qualitative interviews with 20 patients with inoperable lung cancer. Spiritual concerns were important for many patients, both early and later in the progression of the illness. Whether or not patients held religious beliefs, they expressed needs for love, meaning, purpose and sometimes transcendence.

According to O'Driscoll et al.,^8^ breathlessness is a common problem in advanced cancer. It is ranked among the ten most common symptoms among patients admitted to palliative care units. These authors reported that, during conversations with 52 patients with lung cancer, both physical and emotional sensations were associated with descriptions of breathlessness, such as feelings of being unable to get enough breath, or with descriptions of panic or impending death. Ekfors and Petersson^9^ described the experiences during radiotherapy of patients suffering from lung cancer, using a qualitative study design. They found that the patients’ experiences fell into four categories: fatigue, physical distress, disease and treatment management-related issues, and obstacles to such management. Furthermore, Hopwood and Stephens,^10^ evaluating self-reported depression rates among patients with inoperable lung cancer, encountered self-rated depression in one-third of these patients before treatment, which persisted in more than 50% of them.

From a literature review covering 25 years, Montazeri et al.^11^ concluded that the most diffi-cult problem in quality-of-life studies came from the many methodological issues such as data collection, analysis and barriers to the interpretation of the results. The role of the family, relatives, social life, economics and leisure time received relatively little attention in such quality-of-life investigations. According to these authors, focusing only on disease or treatment-related symptoms made these quality-of-life studies very limited. The authors believed there was an urgent need to investigate such issues more comprehensively, since lung cancer patients had indicated that family or leisure time issues were as important as their health. They believed that understanding lung cancer patients’ feelings and concerns might help to improve both the quality of the care received and their quality of life. There was evidence that patients did not necessarily share clinicians’ priorities or place the same emphasis on different types of morbidity.

Recently, Chapple et al.^12^ designed a qualitative study to draw on narrative interviews with patients with lung cancer and also to explore their perceptions and experience of stigma. These authors observed that the participants experienced the feelings commonly felt by patients with other types of cancer. However, whether they smoked or not, they felt particularly stigma-tized because the disease is so strongly associated with smoking. Their interactions with family, friends and doctors were often affected as a result, and many patients, particularly those who had stopped smoking years ago or had never smoked, felt unjustly blamed for their illness.

Therefore, we believe that our present research is of fundamental interest for health professionals who deal with patients with lung cancer. An understanding of the psychodynamics, especially the ego defense mechanisms, and of the role attributed to cigarettes, not only allows for better support, but also for better doctor-patient relationships. This gives rise to improvements in clinical symptoms, which are translated into better quality of life.

## OBJECTIVE

The aim of the present study was to interpret the meanings that patients with lung cancer who were smokers attributed to the possible causes of their diseases, and to understand their perceptions relating specifically to cigarette use.

## METHODS

This study had a clinical-qualitative design. Thus, it adopted a humanistic model, in seeking to scientifically interpret the meanings that individuals’ life experiences acquire, considering these persons’ natural settings. Hence, the present work had an exploratory, non-experimental character.

If one wants to explain asthma scientifically, this is a matter for researchers of pulmonary diseases, immunology, and so on. But if one wishes to understand what asthma means for the patient's life, this is a matter for qualitative researchers. These can be psychologists, psychoanalysts, sociologists, anthropologists or nurses. But it is extremely useful for physicians themselves to make use of qualitative methods. Through their professional experience, they bring in clinical and existentialist attitudes that enable them to perform both valuable data collection and authoritative interpretation of the results. Qualitative researchers study things in their natural settings, in an attempt to interpret phenomena in terms of the meanings people place on them.^13^ Such methods have their own characteristics relating to sample composition, data analysis and the possible generalizations from the results.^14^

The specific strategy utilized in the present research was the so-called clinical-qualitative method. This is considered to be a particularization and refinement of the generic qualitative methods of human sciences, which here was applied to a healthcare setting.^15^ The data collection instrument was the so-called semi-directed interview with open-ended questions. This approach had the aim of ensuring that the matter was discussed with the interviewees in depth.^16^ It has been proved to be appropriate for qualitative research within the field of healthcare, as shown in the literature.^17–19^

The sampling method utilized for qualitative research does not require statistical representativeness in relation to the subject population, i.e. it does not require the use of randomized studies. The procedure involves intentionally seeking out individuals who possess information on the matter to be focused on and the characteristic of articulateness. This produces data with the aim of reformulating, deflecting, complementing and/or clarifying initial hypotheses, as is desirable in any scientific construction.

The study sample consisted of 11 patients with lung cancer. The sample was closed at this number by utilizing the saturation criterion. Thus, it was considered that the incorporation of additional interviews would make little significant contribution with regard to the objectives initially considered for this study. The transcriptions from the interviews formed the corpus for the study and were subjected to qualitative content analysis. Free-floating readings of the interviewees’ responses had been made, so that the researchers would be able to familiarize themselves with the material.

After applying the categorization strategy, the categories for this study were selected. Qualitative analysis of a text does not infer categories from the frequencies of the analysis units (or from other mathematical approaches). Inductive reasoning stemming from identifying the phenomena associated with the interviewees’ responses is utilized. The phenomena thus identified can then be interpreted so as to generate concepts capable of generalization to other settings. The topics thus delineated in the present study were discussed and interpreted according to psychodynamic concepts that are customarily applied within the discipline of medical psychology. The method adopted follows the traditional medical curriculum, in which the teaching of such discipline comprehends a theoretical framework that encloses an interdisciplinary approach in medicine, in which there is a work among psychologists, psychoanalysts and health professionals in order to both qualitatively understand and manage medical matters, such as difficulties in doctor-patient relationship and psychosomatic disorders.

The interviews were conducted among inpatients from the Pulmonary Disease Ward of the General Hospital of Universidade Estadual de Campinas (Unicamp) inside the rooms within the ward. With the help of the nursing team, a strategy was devised to ensure privacy for the interviewee, so as to establish an empathic, cordial and confidential relationship between subject and researcher. The following criteria for selecting patients were established, such that patients were only included if they presented:

- Confirmed diagnosis of lung cancer from the respective medical team;
- Clinical, emotional and intellectual conditions that made them capable of undergoing a clinical-psychological research interview;
- Agreement to their participation, expressed through a statement declaring their free and informed consent, in accordance with the approval for the study granted by the Ethics Committee of the Institution.

The following criteria were not used for patient inclusion/exclusion: gender, age, origin, marital status, family composition, educational level, socioeconomic status and religion. Nevertheless, in order to deal properly with possible bias, the variations in these factors were taken into account in interpreting the results.

## RESULTS AND DISCUSSION

The sample profile was built up from qualitatively representative individuals. They came from a variety of biodemographic groups and presented different psychosocial characteristics. We interviewed eleven patients with lung cancer, with the following features listed in Table 1: ages ranging between 46 and 68 years; six males and five females; some subjects presenting a stable clinical condition and others worsening through incapacitating sequelae. Some of the subjects were still awaiting anatomopathological confirmation results, but had imaging findings consistent with lung neoplasia and were already undergoing chemotherapy, radiotherapy or surgery, according to each specific case. All of them were smokers.

**Table 1.** Sample characterization by individual: gender, age, marital status, profession, working status, length of dependence on cigarettes, clinical diagnosis and treatment

Individual	Characteristics
1	Male, 65 years old, married, retired taxi driver. Tobacco dependence for 39 years, lung neoplasia without anatomopathological confirmation. Chemotherapy and radiotherapy.
2	Female, 58 years old, married, retired domestic maid. Tobacco dependence for 35 years. Lung neoplasia without anatomopathological confirmation. Thoracic drainage, awaiting clinical decision.
3	Female, 68 years old, widow, retired farm worker. Tobacco dependence for 40 years. Lung neoplasia without anatomopathological confirmation. Chemotherapy.
4	Male, 49 years old, married, retired metalworker. Tobacco dependence for 27 years. Non-oat cell carcinoma. Chemotherapy, awaiting radiotherapy.
5	Male, 52 years old, single, retired driver. Tobacco dependence for 30 years. Non-oat cell carcinoma compatible with epidermoid carcinoma. Chemotherapy, awaiting radiotherapy.
6	Male, 46 years old, married, retired mechanic. Tobacco dependence for 30 years. Adenocarcinoma. Surgery (lobectomy), chemotherapy, radiotherapy.
7	Male, 58 years old, married, retired construction worker. Tobacco dependence for 30 years. Oat cell carcinoma. Chemotherapy.
8	Male, 66 years old, single, retired plumber. Tobacco dependence for 60 years. Squamous cell carcinoma. Chemotherapy.
9	Female, 61 years old, widow, retired farm worker. Tobacco dependence for 53 years. Non-oat cell carcinoma compatible with adenocarcinoma. Awaiting clinical decision.
10	Female, 51 years old, divorced, retired farm worker. Tobacco dependence for 3 years. Non oat-cell carcinoma. Chemotherapy.
11	Female, 58 years old, married, domestic maid. Tobacco dependence for 40 years. Adenocarcinoma. Surgery (lobectomy).

### Cigarettes and pleasure

Although most of these smokers seemed aware of the damage to health that tobacco use causes, this had not led them to give up smoking. Several factors are implicated in the smoking habit, and among these is the pleasure obtained. The interviews indicated that cigarettes invoked unique sensations and experiences, which were probably caused both by the action of nicotine on the central nervous system receptors, in association with a sensation of reward, and by the representation given to the patients’ self-image and social image. In our culture, taking a drag on a cigarette is attractive and sensual. In the interview material, the idea of status associated with the smoking habit was clearly noted. It brought an escape from stressful reality and was a pleasant thing giving the feeling of happiness. Nonetheless, these human feelings and attitudes are normally marked by ambiguousness.

*“When I saw the cigarette, I took a drag. What a delight! I don't know how many times I had to hold onto the coffee tree trunk so I wouldn't fall. It was so strong! That's how it was… I even remember the cigarette brand I started with, the one that the boy taught me to take my first drag on. That first drag; when I started, I almost threw up (…) Until I got how to do it. (…) Those girls arrived there and I pulled out the cigarette pack. I took a few drags and I thought I was doing great, right?”* [Patient 6]

The start of consumption was generally described as a moment of discovery during childhood or adolescence, infused with a certain innocence. This reference seemed to minimize the negative sense attributed to cigarettes, as a vice and addiction.

*“It was a problem of being one of the lads. I started out as a bad lad and went on like that until I felt it was making me sick.”* (Patient 1)

*“As a girl from the country, I had my vacations at my grandmother's home, on a farm. That's when I saw people smoking. I thought it was nice, you know. I started for fun; then the vice of cigarettes got very difficult…”* [Patient 11]

It became difficult for the patients to question this addiction, when they also considered it as something that was pleasurable, or even to change the way they psychologically rationalized their smoking, over the short term. When they were asked about the causes of their lung cancer, several interviewees attributed their disease to factors other than cigarettes. Since cigarettes were a source of gratification and pleasure in the smokers’ lives for a long time, it became almost impossible to condemn them unequivocally as largely responsible for their disease. Allowance was always given for the object of their love.

### Attribution of cancer to previous diseases previous diseases

The patients’ cancer outcome was repeatedly attributed to other supposedly causal pulmonary disorders that had occurred previously, such as pneumonia, bronchitis, tuberculosis and emphysema. In the patients’ perception, their cancer represented fragility and impotence. In the fantasy created by these ill individuals, strong and healthy bodies were immune to any type of morbidity. Strong people would never get sick. Following this line of reasoning, if the lungs sickened seriously, then these organs had already suffered previous attacks and the demands on its functions had led to exhaustion.

*“I think that it was caused; well, it even happened because of bronchitis, didn't it? I believe that it was caused by bronchitis, because I got back my strength to cough, didn't I?”* [Patient 3]

*“I caught tuberculosis, after pneumonia, because of my sedentary work. I worked in the open air. (…) and then it got better, but the complications remained (…). Pneumonia, bronchitis, pulmonary emphysema… (…).”* [Patient 5]

### Cancer attributed to mystical factors

In addition, some of the interviewees’ responses attributed the appearance of cancer to superhuman factors, manifested through magical or magical-religious concepts (and hence, non-scientific ideas), such as the will of God or punishment of Evil. Cancer would come, for example, as a divine ordainment to test the patient's faith.

It is known that people customarily create a kind of lay theory in the form of a set of ideas, through voluntary or involuntary mental mechanisms. This may come from both the inner world (unconscious fears) and the outer world (cultural beliefs). The ordering of these ideas aims to meet the particular psychodynamic and psychocultural needs of people with health problems, for them to cope with the phenomena of the health-disease process.

*“I believe that, with the little that God has for me, the ‘other’ doesn't pass by, does it?”* [Patient 2]

*“The truth is that it's God who would give this disease (…). People say that it's the devil; that God does not give this illness to us. God wouldn't give us anything bad.”* [Patient 9]

### Attribution of cancer to the smoking habit

Finally, there were some patients who clearly attributed the cause of their disease to their smoking habit. However, we noted that in these cases their assimilation of the medical explanations was poor. These individuals demonstrated an implicit feeling of intense guilt. The way that patients are informed about the etiological factors in diseases, through the scientific-natural model, may in itself become anxiogenic for them. They thus perceive themselves to be carriers of carcinogenic agents. For self-preservation and to face up to the disease, such individuals involuntarily seek ego defense mechanisms such as rationalization and intellectualization.

*“The day I felt that it was making me sick was when I went to see the doctor (…) He joked with me, and I almost had a fight with him. I stopped smoking on that day; ever since that day. I've never again held a cigarette in my hand. But what I'm feeling today is the legacy of cigarettes. For sure.”* [Patient 1]

*“According to the doctor, it was because I was smoking too many cigarettes (…) I had thought that sometimes cigarettes don't make anyone sick. Later on, I saw for myself that it was the cigarettes, and I had to stop smoking because I couldn't bear to smoke any more. I had to stop, and I recognized that it was cigarettes that destroyed my lungs and me…”* [Patient 8]

Although the attribution of this disease to tobacco is scientifically recognized, it obviously cannot be said that this is an absolute generalization. On certain occasions, the patients used this concept that there might be exceptions, in order to refute the medical finding that tobacco is the greatest cause of lung cancer. In this, their views coincided in some points with the findings by Chapple et al.^12^ On this basis, they needed to formulate other hypotheses to explain the disease occurrence, albeit with low plausibility.

*“My doctor thinks that this was due to smoking, and now I think all doctors believe so. I've been smoking for many years and they suspect that cigarettes are responsible for it (…). Why are there so many people who smoke, who smoke even more than me, and don't have any problem? I think that it's destiny, let's put it like that. It's someone's destiny. They've got to get through that phase…”* [Patient 7]

There were some statements in which the interviewees blamed cigarettes for their disease, but they emphasized that their addiction started because of stimulus from other people, generally relatives, who functioned as inappropriate role models. These patients tended to take on the position of victims, alleging that at that time they did not have enough maturity and discernment to resist smoking.

*“At that time, my father already smoked those big cigarettes… So, he was providing stimulation to those little children that were coming, to the next generation… If he had said: ‘Ah, my son, do not do this; this is foolish, this is poison…’ But he never told me this! He was the teacher and I was the victim. And I fell into the trap.”* [Patient 6]

Despite everything, it was possible, in some parts of the interviews, to recognize non-pathological feelings of guilt. In these, the patients’ personal experiences of the drama of the disease allowed them to speak about the relationship between cigarettes and cancer. There were some patients who seemed to wish to emotionally elaborate regarding the presence of the cancer:

*“I thought that it was deceitful advertising, you know. Perhaps it was advertising by someone who does not smoke. But today, I really see that it was not so (…) I needed to come here to the hospital to be able to believe that it was this that really made me sick.”* [Patient 11]

Our results regarding the attributions of the causes differed partially from what was found by Faller et al. In these authors’ work, the factors most commonly associated by patients with lung cancer were both cigarette consumption and the presence of toxins in their patients’ working environment.^20^ How-ever, just as in our results, they found that patients frequently minimized the harmful action of cigarettes, through trying to alleviate their strong guilt.

### Condemnation of tobacco

Despite the reasons that the patients gave for why they had the disease, they were practically unanimous in affirming that cigarettes caused some degree of harm. They thus felt that they had an obligation to alert society to the risks that are hidden inside the “harmless” cigarette package, the passion for the glamour of smoking and the “innocent” addiction from an early age. Because they had loved smoking, and because they now hated their diseased state and faced guilt, their psychological attitudes of both reparation and sublimation remained.^21^

*“I need to pass on a little of the experience I've had, you know? What I could do? What had to happen, happened. There's no return. So, I'd like people to use me as an example so that they don't do the same thing. It's very easy for people to say: Ah, you must not drink; you must not smoke. (…) Just telling someone is no good. I had to get to this point to be able to see the point. It's not easy, not at all easy!”* [Patient 11]

## CONCLUSION

One of the hypotheses initially considered in this project, i.e. that patients would attribute their disease to cigarette consumption, must be partially reviewed. The subjects often tried to minimize the causal impact of cigarettes and questioned their real influence, even though they had been aware of their diagnoses for a long time and were hospitalized at that moment. To change their views through feelings of guilt would lead to excessive conflict and, thus, too much anguish for the patients to openly admit their responsibility for having fostered their own disease.

On the other hand, medical explanations may, even involuntarily, aggravate the patients’ negative feelings such as guilt and worthlessness, through suggesting that lung cancer is a self-inflicted disease. The philosophy of natural sciences, which internal medicine forms part of, breaks down with the psychosocial/cultural complexity involved in drug consumption, for epistemological reasons. Drug consumption involves phenomena within the individual's psychological development, related to conscious and unconscious motivations, social-environmental and educational factors, physical and psychological dependence, and the relationship with the glamour of tobacco advertising.

Although the information revealed is all theoretically important for gaining a clinical understanding of the disease, it must be underlined that there is an iatrogenic risk in the direct conversation approach. Overall, because such patients are in a phase of both existential fragility and psychological regression, caused by a disease that still carries stigmatism, doctors and nurses can be perceived as “inquisitor figures”. This may complicate the patient's adherence to treatment and follow-up.

Finally, in some extreme situations, certain perceptions regarding the smoking habit were so associated with great craving for cigarettes that the psychological pleasure from smoking was described as “emptied”, probably because of great physical dependence on nicotine.

In addition to this, we recommend that health professionals should take on an attitude of giving clinical value to the significance that such patients place on the phenomena of deep psychological links with cigarettes and their fantasies regarding the possible causal factors for their cancer. Knowing what people with such diseases imagine and feel facilitates the doctor-patient relationship, and also brings a wealth of material for public health campaigns, for example. There is a need to inform both patients and the general population regarding primary and secondary prevention measures, but this is not enough. It has been said that, if scientific information were enough, there would not be any physicians who smoked. It is essential to know what things mean for people, because meanings have a crucial structuring function for both individuals’ and the community's lives. We organize our lives around what things mean for us, including our healthcare.
